# Item Generation in the Development of an Interactive Nutrition Specific Physical Exam Competency Tool (INSPECT): A Qualitative Study Utilizing Technology-Based Focus Groups in the United States

**DOI:** 10.3390/healthcare9050576

**Published:** 2021-05-13

**Authors:** Sunitha Zechariah, Leigh Lehman, Jennifer L. Waller, Gianluca De Leo, Judith Stallings, Ashley J. Gess

**Affiliations:** 1 Applied Health Sciences Program, College of Allied Health Sciences, Augusta University, Augusta, GA 30912, USA; 2 Morrison Healthcare, Sandy Springs, GA 30350, USA; 3 School of Occupational Therapy, Brenau University, Norcross, GA 30071, USA; llehman@brenau.edu; 4 Department of Population Health Sciences, Division of Biostatistics and Data Science, Augusta University, Augusta, GA 30912, USA; jwaller@augusta.edu; 5 Department of Interdisciplinary Health Sciences, College of Allied Health Sciences, Augusta University, Augusta, GA 30912, USA; gdeleo@augusta.edu; 6 Department of Physician Assistant, College of Allied Health Sciences, Augusta University, Augusta, GA 30912, USA; jstallin@augusta.edu; 7 Department of Teaching and Leading, College of Education, Augusta University, Augusta, GA 30912, USA; AGESS@augusta.edu

**Keywords:** nutrition-focused physical exam, competency, focus groups, item generation, malnutrition, registered dietitian nutritionist

## Abstract

An alarming 30% to 50% prevalence rate of disease-related malnutrition among hospitalized patients compels the need for early diagnosis and treatment of malnutrition. Registered Dietitian Nutritionists (RDNs) can utilize the nutrition-focused physical examination (NFPE) as one of the nutrition assessment criteria to accurately diagnose malnutrition. Although RDNs are striving to employ NFPE in practice, a lack of experience and adequate training impedes full utilization of this technique. This results in wide skill variations requiring continuous evaluation of RDNs’ NFPE competency. However, a standardized, validated competency tool is not widely available and hence this study aims to develop a standardized, interactive nutrition-specific physical exam competency tool (INSPECT). As a first step in the development of INSPECT, a qualitative, technology-based focus group approach with 7 content and practice experts was utilized to generate appropriate tool items. A total of 70 NFPE items under 9 areas including 12 items for muscle loss, 4 items for subcutaneous fat loss, 31 items for micronutrient deficiencies, 1 item for fluid status, 2 items for handgrip strength, 5 items for initial preparation, 4 items for bedside manner, 8 items for swallowing, and 3 items for abdominal evaluation were generated. This study successfully utilized technology-based focus groups to generate appropriate NFPE items for the competency tool development. Using the items, an initial version of INSPECT has been developed, which is presently being investigated for content and face validity. The final version will undergo field tests and will be examined for reliability, validity, and item-level psychometric properties.

## 1. Introduction

Disease-related malnutrition is a growing concern in today’s healthcare landscape with an estimated prevalence rate as high as 30% to 50% among patients hospitalized in the United States [1,2,3]. The mean rate of prevalence based on a retrospective review of 20 studies has shown to be a substantial 41.7% [1]. Malnutrition has devastating effects, particularly in patients with chronic disease conditions. It impairs recovery; is associated with high morbidity and mortality rates; results in poor clinical outcomes and functional status decline [4,5,6,7,8,9,10,11]. In addition, malnutrition imposes an enormous economic burden, with disease-associated malnutrition costs totaling $157 billion in the United States [12]. Such a high rate of prevalence along with serious health and economic implications calls for early detection and accurate diagnosis of malnutrition along with timely treatment.

Despite the disconcerting prevalence and consequences of malnutrition, only about 4% to 8% of the hospitalized patients are actually being diagnosed and documented as malnourished [2,13]. Tobert and colleagues (2018) analyzed 5,896,792 hospitalizations across 105 hospitals in the United States over a 2-year period and found that the median documented rate of malnutrition to be 4% with a range of 0.65% to 18.6% [2]. This huge discrepancy between the rate of prevalence and the rate of diagnosed malnutrition is due to underdiagnosis [14] and under-documentation of malnutrition in clinical practice. When researchers dedicate the time to study the prevalence of malnutrition, alarming rates are identified. In routine clinical practice, however, the majority of cases of malnutrition are underdiagnosed, untreated, and under-documented [15,16].

One of the major reasons for underdiagnosing malnutrition is that there is no single objective marker or laboratory test for diagnosis [2,17,18]. For decades, healthcare providers, including Registered Dietitian Nutritionists (RDNs) have underdiagnosed malnutrition due to dependence on various laboratory markers, nutrition screening tools, and devices such as dual-energy X-ray absorptiometry and bioelectric impedance analysis [14,17]. In 2012, to address the need for a standardized approach to diagnosing malnutrition, the nation’s two most reputable professional nutrition organizations, the Academy of Nutrition and Dietetics (the Academy) and the American Society of Parenteral and Enteral Nutrition (ASPEN) collaborated to provide a diagnosis framework [11,19]. This collaborative effort resulted in a consensus statement that included 6 diagnostic criteria to identify all types of adult malnutrition. The criteria include insufficient oral intake, weight loss, loss of muscle mass, loss of subcutaneous fat, localized and/or generalized fluid accumulation, and functional handgrip strength [11,19]. Of these 6 characteristics, food intake and weight loss are most often obtained through nursing or patient/caregiver reports and medical documentation. The remaining 4 characteristics of loss of muscle mass, loss of subcutaneous fat, accumulation of fluids, and hand-grip strength are best determined through a comprehensive nutrition-focused physical exam (NFPE) performed by clinicians, in particular by RDNs [20].

The NFPE is a systematic examination of the physical and functional capabilities of patients to assess their nutritional status and to determine the presence of any nutrient deficiencies or excesses. Evaluating muscle mass and subcutaneous fat loss as part of a comprehensive physical exam is crucial, as muscle atrophy and decreased subcutaneous fat are well-established indicators of malnutrition [11,17]. During times of severe illness and stress, insulin production decreases in the body while glucagon levels increase stimulating a breakdown of adipocytes and myocytes. Consequently, fat and muscle stores are depleted resulting in a nutrition deficit, which when not compensated may result in malnutrition [21]. By applying the physical examination techniques of inspection and palpation, the RDNs have an opportunity to uncover clues of atrophied muscles and depleted fat stores in patients. Furthermore, RDNs may discover physical signs of micronutrient deficiencies during the NFPE, which otherwise could easily go unidentified and untreated [22]. Findings from the NFPE can then be utilized by the RDNs to compare data from the patients’ historical information to fully assess the patients’ nutritional status and any existing nutrient deficiencies. Subsequently, RDNs would consolidate the gathered information to identify the nutrition diagnosis and provide the appropriate plan of care.

In spite of the evident need for RDNs to diagnose and treat malnutrition, several factors impede diagnosis. Typically, physicians, physician assistants, and nurse practitioners establish a medical diagnosis by performing a physical exam on patients [22]. As these healthcare professionals focus on identifying the immediate medical concern, any underlying malnutrition may go undiscovered. Unlike the physical exam performed by these practitioners, the NFPE performed by RDNs focuses on nutrient-related clinical changes enabling the RDNs to assemble all of the clinical characteristics towards determining the presence and severity of malnutrition including any existing micronutrient deficiencies [14]. Consequently, there is a compelling need for RDNs to incorporate a hands-on physical examination to identify the signs of malnutrition accurately [17], and if malnutrition exists, to provide appropriate treatment recommendations to alleviate the condition [23].

Despite the aforementioned benefits of a physical examination performed by RDNs, this skill has been severely under-utilized in clinical dietetic practice since it was only added to the revised dietitians’ scope of practice within the past 8 years for practicing RDNs and within the past 5 years for dietetic students [24,25,26]. Additionally, in recent studies, RDNs have indicated several barriers to performing NFPE [20,27]. The barriers include a lack of experience and training in NFPE, inadequate time, and the reluctance to physically touch patients [20,27]. Attempts are being made to train RDNs by using simulation models, in-person training, and video demonstrations. However, there is a wide range of skill and comfort level in applying this technique in practice in addition to a lack of a standardized tool to measure the application of NFPE in routine patient care [20,28,29]. Regular evaluation, refinement, and retraining of RDNs’ skills in performing NFPE [14] are necessary to compensate for the infrequent training and the lack of consistent practice. A limited number of NFPE competency tools are available including those developed in-house as part of didactic curriculums [30], the Academy skill development checklist that is accessible only to the Academy workshop participants [20], and a recently published validated competency tool developed based on evidence-based literature [29]. Nevertheless, there are currently no NFPE competency tools that have been developed with the contribution of content and practice experts based on their current NFPE experience in clinical practice. Hence, as a first step in developing a standardized tool, Interactive Nutrition-Specific Physical Exam Competency Tool (INSPECT), this study aims to generate NFPE competency tool items by utilizing the expertise of content and practice experts through technology-based focus group discussions.

## 2. Materials and Methods

As NFPE is a relatively new area for RDNs [28], a qualitative research approach employing focus group discussions with content and practice experts was utilized to explore appropriate items for the development of INSPECT. A focus group technique was desirable as it was less formal, allowed for open, in-depth group discussions with participants exchanging their viewpoints, experiences, and NFPE practice preferences [31,32,33,34]. A technology-based focus group approach was selected for this study where the experts could log into a virtual meeting room to participate in live discussions. Several studies have utilized technology-based focus groups to generate items for measurement tools [35,36]. This online approach allowed experts to participate from around the nation without the need for travel [35]. The Augusta University institutional review board provided exempt approval for this study (# 1319708-2).

Purposive sampling methodology [37] was employed to recruit actively practicing RDNs from across the United States who have expertise in clinical dietetics and in performing NFPEs. A sample size of 5–7 focus group participants has been recommended as adequate for technology-based focus groups [38]. Seven RDNs deemed as experts based on their clinical and NFPE practice experience were identified and invited to participate in the study. The experts provided a representative sample based on the following criteria: 1. they were from a variety of hospitals including teaching and community hospitals; 2. they were from across the United States representing various geographic locations; 3. they had a wide range of clinical dietetic experience; and 4. they had diverse practice skills in NFPE. Each expert was sent an email with the study description along with a SurveyMonkey® participation link to provide informed consent (Survey Monkey Inc., San Mateo, CA, USA). All invited participants provided informed consent and self-selected 1 of 3 focus group sessions based solely on their convenience and availability. Two sessions contained 2 experts and 1 session had 3 experts to allow ample time for in-depth discussions, adequate participation from each expert, and to avoid an environment where the discussion moved rapidly skimming over the NFPE components [38]. All of the focus group meetings were conducted utilizing Zoom^TM^ online platform (Zoom Video Communications, Inc., 2020, San Jose, CA, USA). To preserve participants’ privacy and to allow for full exchange of ideas without distractions, the video feature of the online platform was not utilized during the focus group sessions [36]. Study participants were informed that the discussions were being audio recorded for future data extraction and analysis, and their rights and responsibilities were reviewed.

The principal investigator S.Z., an RDN and doctoral candidate who has been conducting NFPE related research since early 2018, played the role of the moderator for the semi-structured focus group discussions. Six exploratory, open-ended questions were formulated by S.Z. to reveal participants’ expert opinions regarding potential components of the NFPE and the practical aspects of applying NFPE in clinical practice. The involvement of the moderator was limited to taking notes, guiding the groups to different topics, and ensuring all participants were given adequate opportunity to participate in the discussion [39]. Each of the 3 focus group discussions lasted between 60 to 90 min and all experts participated for the entire duration of the focus group session. Discussions were audio-recorded using Zoom^TM^ online conferencing platform.

The recorded focus group discussions were transcribed verbatim excluding all identifiers to maintain participant confidentiality. Member checking was completed by allowing participants to check, edit, and/or elaborate any part of their own words in the transcribed script [40]. All members participated in the member checking process. The verified transcription was then analyzed using NVivo software (QSR International Pty Ltd. Version 11.4, 2017, Melbourne, Australia) to reveal emergent codes and themes from the focus group experts.

In the first cycle of coding, in vivo coding was applied [39,41] and labels were assigned by the principal investigator S.Z. to words and short phrases, establishing a detailed inventory of NFPE components. A second cycle of pattern coding [39,41] was conducted to further refine the initial coding and to consolidate the NFPE elements into categories. This coding and recoding process produced common themes and item pools of NFPE. A co-investigator and one of the project advisors, A.G., independently reviewed and agreed with the recoded labels. NFPE item components and themes identified from the coding and recoding process were discussed over multiple meetings between S.Z. and A.G. to reach consensus, thus improving reliability [39]. These item pools served as the framework for the initial item bank of NFPE components for INSPECT.

## 3. Results

All 7 NFPE experts who were invited in December 2018 agreed and participated in the technology-based focus group discussions, resulting in a 100% response rate. All experts took part in the entire process of this study including the focus group interviews and the member checking process. All participants were females, identified themselves as White, had a median clinical dietetic experience of 22 years (range = 5 to 43 years) and a median NFPE experience of 10 years (range = 2 to 40 years). Participants were employed as clinical dietitians (n = 3, 43%), clinical nutrition managers (n = 3, 43%), or as an educator/researcher (n = 1, 14%). Table 1 depicts participant characteristics and their geographic locations. 

**Figure 1:**
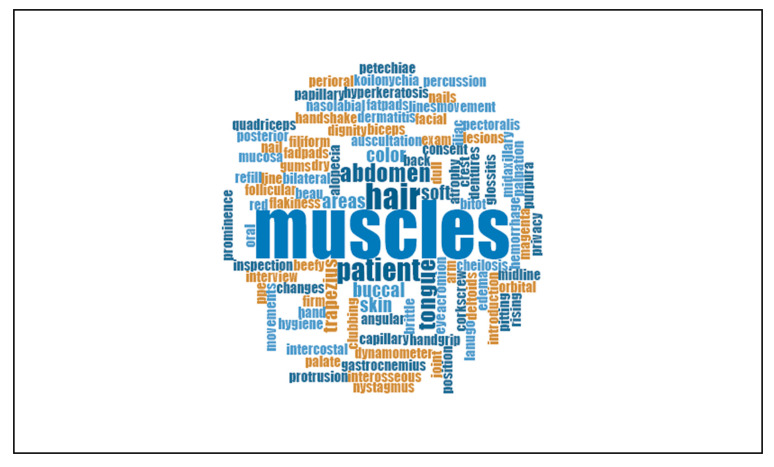
Word Cloud of 70 nutrition-focused physical examination (NFPE) Items Generated from Focus Groups

All participants agreed that NFPE is an important skill and should be incorporated as part of the routine nutrition assessment process by RDNs. All participants also concurred that hands-on NFPE training and competency evaluation should begin at the undergraduate level for dietitians to become adequately proficient to begin practice. The initial in vivo coding analysis of the focus group transcription resulted in 111 NFPE item components. The recoding process allowed for refinement of the item pool to 70 items. The 70 NFPE item components generated from the focus group discussions are presented as a Word Cloud in Figure 1.In addition, two themes emerged from the expert focus group discussions: (1) culturally sensitive evaluation and (2) NFPE competency evaluation of RDNs.

### 3.1. NFPE Item Components

Content experts discussed various components of the physical exam, which are presented here in 9 categories: muscle loss, subcutaneous fat loss, micronutrient deficiencies, fluid status, handgrip strength, basic swallow assessment, abdominal evaluation, initial preparation to NFPE, and bedside manner while performing NFPE.

#### 3.1.1. Muscle Loss

All experts agreed that accurate assessment of muscle loss is a crucial part of malnutrition diagnosis and identified the most frequently inspected and palpated muscle groups during the NFPE exam. Six out of 7 experts agreed that they begin their hands-on assessment focusing on the temporalis muscle. Experts discussed 8 muscle groups in varying order, which comprised of the trapezius, deltoids, pectoralis, scapular (supraspinatus and infraspinatus), interosseous, quadriceps, and gastrocnemius. Although intercostal muscles, thenar muscles and muscles around midaxillary lines were not explicitly mentioned by their names, experts referenced these muscle groups during their discussion. In total, 12 items focusing on muscle loss were identified and the corresponding exemplary quotes from the expert focus group discussions are given in Table 2.

#### 3.1.2. Subcutaneous Fat Loss

Experts largely discussed inspecting and palpating 4 areas of subcutaneous fat loss including orbital fad pads, buccal fat pads, triceps, and fat pads between the last rib and iliac crest. Four items were generated from the focus group discussions and the complementing exemplary quotes are given in Table 3.

#### 3.1.3. Micronutrient Deficiencies

All experts expressed inspecting and palpating for micronutrient deficiencies. The main areas of the micronutrient exam involved hair, face, eyes, mouth/oral cavity, upper extremities including skin and nails, and skin on the lower extremities. In total, 31 micronutrient deficiency items were generated during the expert focus group discussions and the corresponding exemplary quotes are shown in Table 4.

#### 3.1.4. Fluid Status

All participants discussed assessing pitting edema in the pretibial area, ankles, and feet. Participants did not discuss any other type of fluid status assessment. Therefore, 1 item of fluid assessment was generated, and the related exemplary quotes are given in Table 5.

#### 3.1.5. Hand Grip Strength

The utilization of grip strength was mixed among the participants. Some experts used grip strength assessment using a hand dynamometer while others used handshake to assess grip strength. Some participants omitted grip strength and instead limited their assessment to interviewing patients on functional activities. Two items were generated for grip strength from the expert discussions and the complementing exemplary quotes are displayed in Table 6.

#### 3.1.6. Basic Swallow and Abdominal Exam

While 2 of the experts considered assessing the abdomen and conducting a basic swallow exam as important components of NFPE, other experts agreed that it is a valuable skill for the RDNs, however felt these components are advanced skills, and they did not routinely apply them in their own practice. In view of developing a competency tool that would cater to the needs of a broad base of RDNs, it was decided to incorporate these items within the tool. Hence, 8 items for the basic swallow exam and 3 items for the abdominal exam were generated and the related exemplary quotes are shown in Table 7 and Table 8 respectively.

#### 3.1.7. Appropriate Preparation and Initial Steps

As part of the initial preparation for NFPE, experts considered hand hygiene, personal protective equipment such as gloves, obtaining patient consent, maintaining patient privacy, and self-introduction as essential steps to the process. Therefore, 5 items were generated to be included as initial steps and the corresponding exemplary quotes from the participants are given in Table 9.

#### 3.1.8. Bedside Manner and Etiquette

The experts discussed bedside manner as a vital part of the NFPE process. Maintaining patient dignity at all times during the exam, performing the exam bilaterally, and ensuring comfort and position of the patient were discussed as essential etiquette in NFPE. In addition, participants highlighted patient interviews as an indispensable component of the NFPE process. In total, 4 items on bedside manner and etiquette were generated from the focus group discussions and the related exemplary quotes are shown in Table 10.

### 3.2. Themes

In addition to the NFPE components, 2 themes emerged from the focus group discussions as critical to the NFPE process. These included culturally sensitive evaluation and evaluating RDNs’ NFPE competency on an ongoing basis.

#### 3.2.1. Theme 1: Culturally Sensitive Evaluation

Only 1 of the 7 experts raised the importance of race and ethnicity while performing NFPE. Since there was no consensus among the experts on this topic, items were not generated from this theme and the exemplary quote is given in Table 11.

#### 3.2.2. Theme 2: NFPE Competency Evaluation of RDNs

Experts agreed that it is critical to evaluate RDN competency at regular intervals to maintain and improve their NFPE skills. Six out of 7 experts agreed that annual or alternate year evaluation was appropriate. Experts also concurred that the direct manager usually completed the evaluation. As this theme was not directly related to performing NFPE, no items were generated from this theme and the complementing exemplary quotes are given in Table 12.

In summary, 70 NFPE items under 9 areas were generated from the expert focus group discussions and are given in Table 13. These items comprised of 12 items for examining muscle loss, 4 items to assess the subcutaneous fat loss, 31 items to evaluate micronutrient deficiencies, 1 item to assess fluid status, 2 items to gauge handgrip strength, 5 items for initial preparation to NFPE, 4 items for bedside manner, 8 items for basic swallowing and 3 items for abdominal evaluation.

## 4. Discussion

The findings of this study offer significant knowledge on the potential competency tool items that are critical to evaluate NFPE skills among practicing RDNs. As there is a paucity of standardized and validated NFPE competency tools [28], this preliminary information will aid in developing INSPECT and other similar NFPE competency tools and will assist in establishing their validity and reliability. To the knowledge of the investigators, this study is the first to engage content and practice experts in in-depth focus group discussions to generate NFPE competency tool items. Allowing the content experts to freely exchange ideas in a non-threatening, open discussion forum stimulated dynamic discussions of similarities and differences in performing NFPE. Such an open, qualitative approach has been successfully applied in several previous studies for item generation [42,43] and in specific, technology-based focus groups have been employed to generate items for tool development [36,44].

Nine areas of NFPE were gleaned as important parts of NFPE competency from the expert focus group discussions: muscle loss, subcutaneous fat loss, micronutrient deficiencies, fluid status, handgrip strength, basic swallow assessment, abdominal exam, initial preparation before NFPE, and bedside manner during NFPE. In total, 70 NFPE tool items were generated to represent these 9 areas. The items generated from the focus group discussions were compared with evidence-informed literature, which revealed the items to be pertinent for NFPE assessment and therefore are considered appropriate items for the development of the INSPECT [14,19,22,23,28,45].

Along with inspection and palpation, experts emphasized the importance of patient interviewing as part of the NFPE process. They resoundingly agreed that it is not only sufficient to inspect and palpate the patients during a physical exam but to also ask pertinent questions to correlate with the findings of the exam [14,22]. Moreover, experts discussed the value of ongoing evaluation of NFPE competency for RDNs to maintain their skill set in clinical practice. Experts suggested a yearly evaluation or evaluating alternate years at a minimum as optimal and recommended direct managers to be responsible for this skill assessment. The Accreditation Council for Nutrition and Education (ACEND), the accrediting agency for dietetic education programs, has recently conducted a competency gap analysis as part of the future education preparation for RDN practitioners and has identified NFPE as one of the required competencies to complete a comprehensive nutrition assessment [24,25]. Therefore, demonstrating adequate competency of this required skill warrants initial NFPE evaluation and ongoing evaluation thereafter, utilizing a standardized competency tool.

Although this study produced spirited expert exchanges, some gaps in discussions were also observed. One main gap noted was the lack of discussion around ethnic and gender identity differences influencing NFPE. Unquestionably, race, ethnicity, gender identity, and gender transition affect the physical exam and the interpretation of its results. In particular, the varying skin color of different ethnic groups means the skin examination of lighter-skinned individuals will differ vastly when compared to darker-skinned individuals [46]. In addition, facial enhancements such as Botox^®^ may modify the appearance and texture of the skin, thus altering the results of the facial exam. However, ethnic variations and cosmetic skin enhancements influencing NFPE were not adequately discussed during the focus group sessions indicating the need for RDNs to be cognitive of these differences and to be able to adjust their physical exam accordingly.

As transgender individuals may opt to transition from one gender to the other using hormonal therapy and/or through gender-affirming surgeries, these practices may have a marked impact on their nutritional status, weight, and body habitus, which in turn, may impact the NFPE performed on these individuals [47,48,49]. Nonetheless, gender identification differences were not discussed during the expert focus group discussions, suggesting that this may be a relatively new area for the RDNs. Increasing awareness and training focused on transgender-centered care is essential for RDNs.

Limitations of this study are the small sample size of the content experts and that they were all White females. It would have been valuable to have included additional experts from diverse gender and ethnic backgrounds. Despite these limitations, the experts were representative as they were from across the United States, representing various geographic locations, had a wide range of clinical dietetic experience and current NFPE practice experience.

## 5. Conclusions

Currently, there is limited availability of standardized and validated competency tools to measure NFPE competency skills among RDNs in clinical practice. An NFPE competency tool such as the INSPECT, which is scientifically developed and rigorously tested for validity and reliability is essential to evaluate RDNs’ competency in performing NFPE on patients. Ongoing competency evaluation will equip RDNs in maintaining and improving their NFPE skills, which in turn will improve their ability to accurately diagnose malnutrition and to provide early patient intervention. As a first step in competency tool development, this study generated a set of 70 NFPE items under 9 areas through expert focus group discussions. The generated items were found to be relevant when validated using evidence-informed literature. These generated NFPE items were used to design and develop the initial version of the INSPECT, which is presently undergoing face and content validity testing. The final version of the INSPECT will be field-tested and assessed for inter-rater and intra-rater reliability and construct validity. Item–response theory methodologies will also be applied to examine the item-level psychometric properties. The resultant standardized and validated tool will be made widely available to evaluate the initial and ongoing NFPE competency among RDNs in clinical practice.

## Figures and Tables

**Table 1:** Characteristics of Focus Group Participants

Participant Code	Job Profile	Gender	Ethnicity	U.S. Geographic Location	Clinical Experience (Years)	NFPE Experience (Years)
JF1	RDN *	Female	White	Ohio	20	8
N2	CNM ^	Female	White	Nebraska	43	10
T3	CNM	Female	White	Oregon	40	40
K4	CNM	Female	White	Tennessee	10	3
JG5	RDN	Female	White	Nebraska	22	22
R6	Researcher/ Educator	Female	White	New Jersey	25	15
S7	RDN	Female	White	Missouri	5	2

* Registered Dietitian Nutritionist; ^ Clinical Nutrition Manager.

**Table 2:** Expert Exemplary Quotes Extracted Verbatim on Muscle Loss

Expert Code	Exemplary Quotes
*JF1*	*“I focus on from head to toe, first would be the temporal, the temple area, then orbital area underneath the eyes... I usually put gloves on and then I touch their face on both sides, rubbing their, like, temporal area and then I take my fingers underneath their eyes feeling for their bone or fat pad.”*
*N2*	*“We examine the temples and the orbital areas and the buccal areas for any loss.”*
*T3*	*“Okay, I am going to just, kind of, picture a patient. The temporal muscle and then we look around the orbital for any type of fat wasting or the buccal areas.”*
*JF1*	*“Okay. The trapezius, the deltoids, the pectorals. We also look at the triceps, the ribs and then we move on down and look at the quads and calf muscle.”*
*T3*	*“We look to the clavicle and acromion process and the different areas around the rib. First with the patient sitting up, we examine triceps and note that—we know at that time we are kind of looking globally at the patient and also interviewing them. We’re looking at interosseous muscles in their hands, I think. There again, we’re looking for any type of visuals for the ribs showing or not both front and back. And we are moving on down to like the thigh areas if there is any concaving of the thighs. And then the muscle wasting, we’re actually doing the touching to feel the quality of the muscle, floor of the calves as well as we’re checking for edema also especially in the lower extremities…”*
*JG5*	*“….. we would work our way like into the clavicle region, look for any muscle wasting there. From there, we go into the hands, look at the fingernails…. Other things that we will look at will go to the chest region”*
*R6*	*“We’ll do the scapula or the SCM. We will have the person either put their arms out front or push against the wall so we can see muscle tone.”*
*S7*	*“I would add that we do do lower extremities especially looking for edema and muscle mass of the quadriceps and down also to the calf as well. We do lift their leg and have them engage their lower calf muscles to assess for lower leg loss.”*
*JF1*	*“Ribs, The same thing, we move in our hands down the side of their body checking for any muscle loss.”*
*S7*	*“And at that same time we are assessing the muscle engagement in the back and then we do the ribcage at the same time to go around their side.”*
*T3*	*“And there is a technique with you squeeze the first thumb and the first finger, and there is a pad on the inside of the hand. And when you squeeze that you can actually feel that muscle if you do it yourself and use this, probably hard. But when you get people that have problems, they get real mushy kind of muscle there….There is also another muscle that is in… it’s one of the muscles and when you make the okay sign with your thumb and first finger, you can also feel the muscle, it’s on the inside of the circle that’s on the hand. And you squeeze a little muscle in there and you can see if it’s there.”*

**Table 3:** Expert Exemplary Quotes Extracted Verbatim on Subcutaneous Fat Loss

Expert Code	Exemplary Quotes
*T3*	*“…we look around the orbital for any type of fat wasting or the buccal areas.”*
*JF1*	*“So for triceps, I—we have them bend their arm and then we feel underneath their arm and the triceps area and see how much kind of like fat pad they have there or if we can touch finger to finger if there is wasting.”*
*JG5*	*“….any wasting of fat within the chest area and sides near iliac crest…”*
*R6*	*“Definitely, triceps, biceps. All of the fat and muscle that is aligned with the malnutrition consensus statements…”*

**Table 4:** Expert Exemplary Quotes Extracted Verbatim on Micronutrient Deficiencies

Expert Code	Exemplary Quotes
*JF1*	*“So we do look at nails, spoon-shaped nails, any pale nails, poor blanching. We’re looking for deficiencies, iron deficiency or vitamin A or C deficiencies on skin.”*
*N2*	*“For the micronutrient, we focus on the oral, perioral area, the skin. And so we are focusing on the tongue and looking specifically and using a tongue blade checking for filiform papillary atrophy and just looking for the all the other micronutrient deficiencies that might be suggestive or other lesions suggestive of a nutrient deficiency.”* *“Look around and inside the mouth, look at the eyes, the skin, the scalp, look for goiter….”* *“ Look at—we are looking at hair on the arms looking for corkscrew hair or swan neck hair, looking at the nails, checking for all the deficiencies there…..”* *“Petechiae, any type of trauma that you notice, you’re going to get a history on that to see if it’s nutrition related….”* *“…we look—you look for paleness, you look for inflammation, you look for Bitot’s spots……”* *“We look for paleness, we look for color, different colors, cheilosis, angular stomatitis, aphthous ulcer….”* *“…we look for seborrheic dermatitis in the scalp…..”*
*T3*	*“…..open the mouth and look in the mouth and look at the teeth even if it’s nothing more than ability to. And this is how good the teeth are and the gums……”* “…*there is the approach of looking at the hair and looking at the bed and seeing if there is hair on the bed and seeing where hair is and then asking questions about, is your hair falling out?......”* “…*the anorexic look for the hair on the arm because they can have lanugo hair on their back and stuff…..”* “…*also when you get going, you can look at the nails. And nails are really hard and they are more advanced. And the biggies are kind of like Beau’s lines, clubbing……”* *“We find thiamine deficiencies in the nystagmus and different micronutrient things like that.”*
*K4*	*“we also are looking for the color of the eyelid because anemia often will show up and we’ll have any eye pretty pink. A lot of eyelids sometimes have more white, so sometimes could be a sign of anemia..”*
*JG5*	*We look for any signs and symptoms of flaking around the face in the nasal labial area…. we go into the hands, look at the fingernails….”* “*Also looking for koilonychia, I think I’m saying that right with the dip in the nail like a water drop could fit in that nail, flattening of the nails… Another thing that we look for also vitamin C deficiency that there could be a black like splinter hemorrhages underneath the nail.* ”“*We do look for the Bitot’s spots but have only found one case of it in the years that I’ve practiced.* *“We’re looking like sparse hair also.”*
*R6*	*“Looking for ridges on the nails potential for paleness or poor perfusion, which could relate to iron deficiency.”* *“We’ll also look at the labia and the buccal mucosa so inside the lips and the cheek looking for any trauma or ulcers using the tongue depressor and a pen light.”* *“And then teeth, edentulism, occlusion, dentures, do they have them, do they not, do they—if they have them do they use them, that sort of thing.”*

**Table 5:** Expert Exemplary Quotes Extracted Verbatim on Fluid Status

Expert Code	Exemplary Quotes
*JF1*	*“And then at that time, we would also check for edema while we are doing the exam.”*
*JG5*	*“We will also go into the lower extremities to look at edema.”*
*S7*	*“if we have an elderly patient we do kind of focus on the foot area when we’re assessing edema as well.”*

**Table 6:** Expert Exemplary Quotes Extracted Verbatim on Hand Grip Strength

Expert Code	Exemplary Quotes
*JF1*	*“We don’t, but we do ask about activity level whether they’re doing their normal ADLs or if they’ve been in bed or chair for 50% of the day and for how long. So we do and ask about energy level, but we don’t use the strength grips.“*
*T3*	*“Well, the best practice is the grip strength with the dynamometer.”*
*K4*	*“yes, we agree that the dynamometer is the best practice and we have one. Do we hold it around with us? Of course, we don’t, it’s too big and bulky. And so often we don’t have it with us when we’re doing a physical exam.”*
*JG5*	*“we don’t have the meter so I have them squeeze my hand and tell them to squeeze it as hard as they can.”* *“….and also we have got the handshake.”*

**Table 7:** Expert Exemplary Quotes Extracted Verbatim on Basic Swallow Exam

Expert Code	Exemplary Quotes
*T3*	*“..mouth is pretty scary for people but as a beginner and basic competency of looking in the mouth and looking even the teeth for chewing and swallowing and stuff is really valuable.”* *“—and then the tongue is really hard, but at least you can start with the teeth and swallowing and things.”*
*R6*	*“We test to cranial nerve V, which is the trigeminal nerve where we look at the motor function and the sensory functions. 7th cranial nerve, which is all the motor function of the face.”* *“…..definitely include the oral, you know the head and neck region, which also includes assessing for the muscles of mastication and the temporomandibular joint to look at range of motion in the jaw region.”* *“9th and 10th cranial nerves, we test collectively that is looking at the motor function of the palate seeing that the palate elevates, which helps protect the nasopharynx region so you don’t regurgitate food and liquid up through your nose. we’re looking to see if the uvula is in the midline. These are all again in the context of proper eating as well as swallowing risk of dysphagia.”* *“12th cranial nerve, we test for that which is motion of the tongue making sure it’s in the middle.”* *“11th cranial nerve, which has motor function and that’s keeping your—really keeping your head and neck upright so it’s the innervation of the sternocleidomastoid muscles, the SCM, and the trapezius muscle because if you are unable to keep your head neck straight while you’re eating, you have higher risk of dysphagia.”*

**Table 8:** Expert Exemplary Quotes Extracted Verbatim on Abdomen Exam

Expert Code	Exemplary Quotes
*JF1*	*“When I was a nutrition support, we did do abdominal exams. We used the stethoscope, listen for bowel sounds. We do percussions.”*
*R6*	*“In our NFPE training class, we do teach to that (talking about listening to bowel sounds with a stethoscope and palpation and percussions of the abdomen)”*
*T3*	*“In the abdominal part, I, as far as like listening to bowel sounds and all that kind of stuff, I have tried and tried and practiced that and I am not doing it. I can’t get—I can’t hear it. I have never been able to figure out why. And the other thing is, in the acute care setting, because the nurses and the doctors are doing that already and we see their notes”*
*S7*	*“you mentioned you guys do is fantastic but I think that is a little bit more advanced to be able to do some of those. I mean moving the tongue and correlating that too with the ability to form a bolus in their safety, I think there is maybe work to be done in that area and that might be a little bit more advanced, the bedside swallow screening with NFPE and then the percussion and listening to bowel sounds, I feel like that kind of could correlate how we have diet order writing privileges.”*

**Table 9:** Expert Exemplary Quotes Extracted Verbatim on Appropriate Preparation and Initial Steps

Expert Code	Exemplary Quotes
*N2*	*“I am speaking fragmented here but certainly, when in-gloving, there is handwashing or sanitized.”*
*T3*	*“And so there are stages that—one of the things I’m going to say is I would require for everything would be patient permission and privacy….. You are required washing hands or ask act to seek permission, anybody else present, the privacy and then tolerance to exam and that kind of stuff, I would do.”* *“Somehow—and it could be in your introductory part a lot of times but I mean, what we found, I don’t know if you guys found this but like if the dietitians are doing exam they’ll have already done chatty about other stuff and they’ll say, well, it’s okay if I examine you for my nutrition so that is when they’ll see us so that was permission.”* *“And as you come in introduced, shake hand and put left hand on the shoulder that you could see what’s going on the deltoid.”* *“So I was working on the grip strength documentation and I was just called and I thought, oh my God, this needs to be part of standard documentation that the patient had permission. There was permission to do the physical exam, you could say positively and there are people present in this room and are tolerant to the exam.”* *“It’s a courtesy consent. But if you document and let’s say, somebody with the behavioral health issues or other decided that this person can physically abuse them, you have a little bit of stuff on your side there that they gave permission for exam.”*

**Table 10:** Expert Exemplary Quotes Extracted Verbatim on Bedside Manner and Etiquette

Expert Code	Exemplary Quotes
*JF1*	*“I think it’s important that they are able to approach the patient with good bedside manners, talking to the patient and letting them know like what we actually do. I always explain to the patient as a dietitian. We like to look for muscle loss and fat loss especially, if they’ve been losing weight or haven’t been eating well and then let them know that that’s what I’m looking for and that’s why I’m touching you and looking at all these muscles. I think that would be good basic approach.”* *“And with their quads and their calf muscle, we have them bend their knees and we do it on both sides.”*
*N2*	*“We talked about how they approach the patient. We talk about attending behavior just how they approach the patient and perform the physical exam.”* *“…if you’re going to pick up a patient’s hand and move it then return it to where it was. So just being respectful and if patients are covered and you uncover them make sure you cover them back up.”*
*N2* *JF1*	*Yes, just I think that interview and history is…* *They are just as important. (referring to interview and history)*
*T3*	*“First with the patient sitting up, we examine triceps and note that—we know at that time we are kind of looking globally on both sides at the patient and also interviewing them.”*
*JG5*	*“And that’s where your interview also plays into your physical exam. That ….so it’s really a collection, I mean, your whole—it’s a collection of interview and physical exam.”* *well, part of that, we will ask, I mean, are you able to walk to the mailbox? Were you able to last month? Were you able to six months ago? Different questions to assess the daily living. Is that hard-to-touch area? No, but it’s definitely supportive and also we have got the handshake.*

**Table 11:** Theme 1: Culturally Sensitive Evaluation

Expert Code	Expert Exemplary Quote Extracted Verbatim
*R6*	*“I think it’s important to look at skin in the context of race and ethnicity as well as, I know it sounds silly, but Botox. We—if you’ve got somebody that is receiving treatment with Botox, it’s going to affect their skin so we need to kind of keep those things in our mind.”*

**Table 12:** Theme 2: NFPE Competency Evaluation of Registered Dietitian Nutritionists (RDNs)

Expert Code	Expert Exemplary Quotes Extracted Verbatim
*JF1*	*“Recently, we have just moved out to 2 years. We were annually evaluated for competency. Our direct manager evaluates….”*
*N2*	*“I mean, I think, if you have validated that they are competent, I would say, an ideal world would be annually, but realistically, every 2 years as fast as time goes.”*
*T3*	*“And I have not done a competency per se on them with the competency checklist and I feel guilty about it, but just haven’t had time for it…. And also I don’t have an actual checklist of it.”*
*K4*	*“I just want to make sure that our staff knows what they are doing so that when they are going something through the doctor that we are all on the same page, we are doing things very similarly and they are using best practice. So that’s why I want to do the yearly competency to make sure they are competent and that if I get called into and also if I can stand with confidence since they are competent.”*
*JG5*	*“we need to do yearly evaluations to assess that we’re all completing the physical in the same manner and that our skills continue to be fresh.”*
*S7*	*“I agree. I think a year—doing the yearly evaluation process at least and then doing chart audits as well especially if it’s a dietitian learning the skill.”*

**Table 13:** Summary of 70 NFPE Items Generated from Focus Groups

Categories	Number of Items	NFPE Components
Muscle Loss	12	Temporalis
		Trapezius
		Deltoids
		Pectoralis
		Intercostal Muscles
		Muscles Around Midaxillary Lines
		Scapular (Supraspinatus)
		Scapular (Infraspinatus)
		Interosseous
		Thenar Muscles
		Quadriceps
		Gastrocnemius
Subcutaneous Fat Loss	4	Orbital Fad Pads
		Buccal Fat Pads
		Triceps
		Fat pads Between the Last Rib and Iliac Crest
Micronutrient Deficiencies	31	Hair changes
		Dry, Dull hair
		Brittleness and Easily Pluckable Hair
		Seborrheic Dermatitis
		Alopecia
		Flaky Face and Nasolabial Areas
		Pale Conjunctiva
		Bitot’s spots
		Nystagmus
		Denture Use (Ill Fitting)
		Perioral Changes
		Angular stomatitis/Cheilosis
		Aphthous Ulcer
		Changes in Gums and Teeth
		Changes in Buccal Mucosa
		Filiform Papillary Atrophy
		Magenta/Beefy-Red Tongue
		Glossitis
		Skin Changes of Upper and Lower Arms
		Corkscrew Hair
		Lanugo
		Nail Color
		Koilonychia (Spoon Shaped Nails)
		Beau’s Lines (Transverse Lines)
		Splinter Hemorrhage Underneath Nails
		Clubbing of Nails
		Capillary Refill
		Skin Changes on Back/Sacrum (Non-healing wounds)
		Follicular Hyperkeratosis
		Petechiae
		Purpura
Fluid Status	1	Pitting Edema
Hand Grip Strength	2	Handgrip using Dynamometer
		Handshake and/or Grip/Squeeze fingers
Basic Swallow	8	Direct Swallow
		Temporomandibular joint (TMJ) Range of Motion
		Sternocleidomastoid Muscle Resistance
		Facial Movements
		Trapezius Muscle Resistance
		Thyroid Evaluation
		Uvula Midline & Soft Palate Rising
		Tongue Protrusion & Movement
Abdominal Exam	3	Palpation for Softness/Firmness of Abdomen
		Percussion for fluid accumulation of Abdomen
		Auscultation of Abdomen (Bowel Sounds)
Appropriate Preparation and Initial Steps	5	Hand Hygiene
		Personal Protective Equipment
		Patient Privacy
		Self-Introduction & Explanation of NFPE
		Patient Consent
Bedside Manner and Etiquette	4	Bilateral Inspection & Palpation
		Interview Patient
		Patient Dignity
		Position Patient
Total	70	

## Data Availability

The data presented in this study are available on request from the corresponding author. The data are not publicly available to protect the confidentiality and privacy of the study participants.

## References

[1] Norman K., Pichard C., Lochs H., Pirlich M. (2008). Prognostic impact of disease-related malnutrition. Clin. Nutr..

[2] Tobert C.M., Mott S.L., Nepple K.G. (2018). Malnutrition Diagnosis during Adult Inpatient Hospitalizations: Analysis of a Multi-Institutional Collaborative Database of Academic Medical Centers. J. Acad. Nutr. Diet..

[3] Jensen G.L., Compher C., Sullivan D.H., Mullin G.E. (2013). Recognizing malnutrition in adults: Definitions and characteristics, screening, assessment, and team approach. JPEN J. Parenter. Enteral Nutr..

[4] Correia M.I., Hegazi R.A., Higashiguchi T., Michel J.P., Reddy B.R., Tappenden K.A., Uyar M., Muscaritoli M. (2014). Evidence-based recommendations for addressing malnutrition in health care: An updated strategy from the feedM.E. Global Study Group. J. Am. Med. Dir. Assoc..

[5] Gariballa S., Forster S., Walters S., Powers H. (2006). A randomized, double-blind, placebo-controlled trial of nutritional supplementation during acute illness. Am. J. Med..

[6] Milne A.C., Potter J., Vivanti A., Avenell A. (2009). Protein and energy supplementation in elderly people at risk from malnutrition. Cochrane Database Syst. Rev..

[7] Neelemaat F., Lips P., Bosmans J.E., Thijs A., Seidell J.C., van Bokhorst-de van der Schueren M.A. (2012). Short-term oral nutritional intervention with protein and vitamin D decreases falls in malnourished older adults. J. Am. Geriatr. Soc..

[8] Philipson T.J., Snider J.T., Lakdawalla D.N., Stryckman B., Goldman D.P. (2013). Impact of oral nutritional supplementation on hospital outcomes. Am. J. Manag. Care.

[9] Lim S.L., Ong K.C., Chan Y.H., Loke W.C., Ferguson M., Daniels L. (2012). Malnutrition and its impact on cost of hospitalization, length of stay, readmission and 3-year mortality. Clin. Nutr..

[10] Thibault R., Makhlouf A.M., Kossovsky M.P., Iavindrasana J., Chikhi M., Meyer R., Pittet D., Zingg W., Pichard C. (2015). Healthcare-associated infections are associated with insufficient dietary intake: An observational cross-sectional study. PLoS ONE.

[11] White J.V., Guenter P., Jensen G., Malone A., Schofield M. (2012). Consensus statement: Academy of Nutrition and Dietetics and American Society for Parenteral and Enteral Nutrition: Characteristics recommended for the identification and documentation of adult malnutrition (undernutrition). JPEN J. Parenter. Enteral Nutr..

[12] Snider J.T., Linthicum M.T., Wu Y., LaVallee C., Lakdawalla D.N., Hegazi R., Matarese L. (2014). Economic burden of community-based disease-associated malnutrition in the United States. JPEN J. Parenter. Enteral Nutr..

[13] Barrett M.L., Bailey M.K., Owens P.L. (2016). Non-Maternal and Non-Neonatal Inpatient Stays in the United States Involving Malnutrition. www.hcupus.ahrq.gov/reports.jsp.

[14] Fischer M., JeVenn A., Hipskind P. (2015). Evaluation of muscle and fat loss as diagnostic criteria for malnutrition. Nutr. Clin. Pract..

[15] Correia M.I., Hegazi R.A., Diaz-Pizarro Graf J.I., Gomez-Morales G., Fuentes Gutierrez C., Goldin M.F., Navas A., Pinzon Espitia O.L., Tavares G.M. (2016). Addressing Disease-Related Malnutrition in Healthcare: A Latin American Perspective. JPEN J. Parenter. Enteral Nutr..

[16] McCauley S.M., Mitchell K., Heap A. (2019). The Malnutrition Quality Improvement Initiative: A Multiyear Partnership Transforms Care. J. Acad. Nutr. Diet..

[17] Bharadwaj S., Ginoya S., Tandon P., Gohel T.D., Guirguis J., Vallabh H., Jevenn A., Hanouneh I. (2016). Malnutrition: Laboratory markers vs nutritional assessment. Gastroenterol. Rep..

[18] Toulson Davisson Correia M.I. (2018). Addressing the Hidden Burden of Malnutrition for Hospitalized Patients. J. Acad. Nutr. Diet..

[19] White J.V., Guenter P., Jensen G., Malone A., Schofield M., Academy Malnutrition Work Group A.S.P.E.N., Malnutrition Task Force A.S.P.E.N., Board of Directors (2012). Consensus statement of the Academy of Nutrition and Dietetics/American Society for Parenteral and Enteral Nutrition: Characteristics recommended for the identification and documentation of adult malnutrition (undernutrition). J. Acad. Nutr. Diet..

[20] Mordarski B. (2016). Nutrition-Focused Physical Exam Hands-On Training Workshop. J. Acad. Nutr. Diet..

[21] Winkler M.F., Malone A.M., Mahan L.K., Escott-stump S. (2008). Medical Nutrition Therapy for Metabolic Stress: Sepsis, trauma, burns and surgery. Krause’s Food and Nutrition Therapy.

[22] Esper D.H. (2015). Utilization of nutrition-focused physical assessment in identifying micronutrient deficiencies. Nutr. Clin. Pract..

[23] Malone A., Hamilton C. (2013). The Academy of Nutrition and Dietetics/the American Society for Parenteral and Enteral Nutrition consensus malnutrition characteristics: Application in practice. Nutr. Clin. Pract..

[24] Accreditation Council for Education in Nutrition and Dietetics (2016). Crosswalk of Knowledge and Competency Statements for CP, DI, DPD, FDE, IDE Programs.

[25] Accreditation Council for Education in Nutrition and Dietetics (2019). Future Education Model Standards for Accredited Graduate Programs in Dietetics.

[26] Academy Quality Management Committee and Scope of Practice Subcommittee of Quality Management Committee (2013). Academy of Nutrition and Dietetics: Revised 2012 Standards of Practice in Nutrition Care and Standards of Professional Performance for Registered Dietitians. J. Acad. Nutr. Diet..

[27] Stankorb S., Radler-Rigassio D., Touger-Decker R. (2010). Nutrition Focused Physical Examination Practices of Registered Dietitians. Top. Clin. Nutr..

[28] Touger-Decker R. (2006). Physical Assessment Skills for Dietetics Practice—The Past, the Present and Recommendations for the Future. Top. Clin. Nutr..

[29] MacQuillan E.L., Ford J., Baird K. (2020). Clinical Competence Assessed Using Simulation: Development of a Standardized Tool to Assess Nutrition-Focused Physical Exam Skill Competence for Registered Dietitian Nutritionists. J. Nutr. Educ. Behav..

[30] Tyler C., Alnaim L., Diekemper J., Hamilton-Reeves J., Goetz J., Sullivan D.K., Gibbs H.D. (2020). Simulations for Teaching and Evaluating Nutrition-Focused Physical Exam Skills. J. Nutr. Educ. Behav..

[31] Dawson S., Manderson L., Tallo V.L. (1993). A Manual for the Use of Focus.

[32] Kitzinger J. (1995). Qualitative research. Introducing focus groups. BMJ.

[33] Krueger R.A., Casey M.A. (2009). Focus Groups: A Practical Guide for Applied Research.

[34] Rowan N., Wulff D. (2007). Using qualitative methods to inform scale development. Qual. Rep..

[35] James N., Busher H. (2016). Credibility, authenticity and voice: Dilemmas in online interviewing. Qual. Res..

[36] Strout T.D., DiFazio R.L., Vessey J.A. (2017). Technology-enhanced focus groups as a component of instrument development. Nurse Res..

[37] Stalmeijer R.E., McNaughton N., Van Mook W.N. (2014). Using focus groups in medical education research: AMEE Guide No. 91. Med. Teach..

[38] Mann C., Stewart F. (2000). Internet Communication and Qualitative Research: A Handbook for Researching Online.

[39] Miles M.B., Huberman A.M., Saldaña J. (2020). Qualitative Data Analysis: A Methods Sourcebook.

[40] Carlson J. (2010). Avoiding Traps in Member Checking. Qual. Rep..

[41] Saldaña J., Saldana J. (2013). An introduction to codes and coding [Chapter 1 from: The coding manual for qualitative researchers]. The Coding Manual for Qualitative Researchers.

[42] Uy E.J.B., Xiao L.Y.S., Xin X., Yeo J.P.T., Pua Y.H., Lee G.L., Kwan Y.H., Teo E.P.S., Vaingankar J.A., Subramaniam M. (2020). Developing item banks to measure three important domains of health-related quality of life (HRQOL) in Singapore. Health Qual. Life Outcomes.

[43] George J., Phun Y.T., Bailey M.J., Kong D.C., Stewart K. (2004). Development and validation of the medication regimen complexity index. Ann. Pharmacother..

[44] Wong E.L., Coulter A., Cheung A.W., Yam C.H., Yeoh E.K., Griffiths S. (2013). Item generation in the development of an inpatient experience questionnaire: A qualitative study. BMC Health Serv. Res..

[45] DiBaise M., Tarleton S.M. (2019). Hair, Nails, and Skin: Differentiating Cutaneous Manifestations of Micronutrient Deficiency. Nutr. Clin. Pract..

[46] Everett J.S., Budescu M., Sommers M.S. (2012). Making sense of skin color in clinical care. Clin. Nurs. Res..

[47] Linsenmeyer W., Drallmeier T., Thomure M. (2020). Towards gender-affirming nutrition assessment: A case series of adult transgender men with distinct nutrition considerations. Nutr. J..

[48] Rahman R., Linsenmeyer W.R. (2019). Caring for Transgender Patients and Clients: Nutrition-Related Clinical and Psychosocial Considerations. J. Acad. Nutr. Diet..

[49] Klein D.A., Paradise S.L., Goodwin E.T. (2018). Caring for Transgender and Gender-Diverse Persons: What Clinicians Should Know. Am. Fam. Physician.

